# Next-generation sequencing study reveals the broader variant spectrum of hereditary spastic paraplegia and related phenotypes

**DOI:** 10.1007/s10048-019-00565-6

**Published:** 2019-02-19

**Authors:** Ewelina Elert-Dobkowska, Iwona Stepniak, Wioletta Krysa, Karolina Ziora-Jakutowicz, Maria Rakowicz, Anna Sobanska, Jacek Pilch, Dorota Antczak-Marach, Jacek Zaremba, Anna Sulek

**Affiliations:** 1 Department of Genetics, Institute of Psychiatry and Neurology, Sobieskiego 9 Street, 02-957 Warsaw, Poland; 2 Department of Clinical Neurophysiology, Institute of Psychiatry and Neurology, Warsaw, Poland; 3 Department of Paediatric Neurology, Medical University of Silesia, Katowice, Poland; 4 Clinic of Neurology of Children and Adolescents, Institute of Mother and Child, Warsaw, Poland; 5 Division Five of Medical Sciences, Polish Academy of Science, Warsaw, Poland

**Keywords:** Ataxia-spasticity, Hereditary spastic paraplegia, Movement disorders, Next-generation sequencing.

## Abstract

Hereditary spastic paraplegias (HSPs) are clinically and genetically heterogeneous neurodegenerative disorders. Numerous genes linked to HSPs, overlapping phenotypes between HSP subtypes and other neurodegenerative disorders and the HSPs’ dual mode of inheritance (both dominant and recessive) make the genetic diagnosis of HSPs complex and difficult. Out of the original HSP cohort comprising 306 index cases (familial and isolated) who had been tested according to “traditional workflow/guidelines” by Multiplex Ligation-dependent Probe Amplification (MLPA) and Sanger sequencing, 30 unrelated patients (all familial cases) with unsolved genetic diagnoses were tested using next-generation sequencing (NGS). One hundred thirty-two genes associated with spastic paraplegias, hereditary ataxias and related movement disorders were analysed using the Illumina TruSight™ One Sequencing Panel. The targeted NGS data showed pathogenic variants, likely pathogenic variants and those of uncertain significance (VUS) in the following genes: *SPAST* (spastin, SPG4), *ATL1* (atlastin 1, SPG3), *WASHC5* (SPG8), *KIF5A* (SPG10), *KIF1A* (SPG30), *SPG11* (spatacsin), *CYP27A1*, *SETX* and *ITPR1*. Out of the nine genes mentioned above, three have not been directly associated with the HSP phenotype to date. Considering the phenotypic overlap and joint cellular pathways of the HSP, spinocerebellar ataxia (SCA) and amyotrophic lateral sclerosis (ALS) genes, our findings provide further evidence that common genetic testing may improve the diagnostics of movement disorders with a spectrum of ataxia-spasticity signs.

**Electronic supplementary material:**

The online version of this article (10.1007/s10048-019-00565-6) contains supplementary material, which is available to authorized users.

## Introduction

Hereditary spastic paraplegias (HSPs) comprise a group of genetic disorders resulting from neurodegeneration of the corticospinal tracts. The HSPs’ main clinical feature is a progressive spasticity and weakness of the lower limbs. HSP is classified as a pure form when symptoms are limited to: progressive spasticity and weakness of the lower limbs, bladder dysfunction and mild somatosensory deficits. In case of any additional neurological symptoms, a complicated HSP form is recognised. To date, over 70 different SPG loci have been identified, and over 60 corresponding genes have been investigated [1–3]. All modes of HSP inheritance have already been described: autosomal dominant (ADHSP), autosomal recessive (ARHSP), X-linked (XLHSP) and less frequently, mitochondrial. Among 20 different ADHSP subtypes, SPG4 is the most common one, accounting for approximately 40% of the cases. The frequency of other ADHSP subtypes ranges from 1% to 10%. The main ARHSPs identified to date are SPG5, SPG7, SPG11 and SPG15 [4].

According to population studies, the proportion of families without genetic diagnosis ranged from 45% to 67% in the ADHSP and from 71% to 82% in the ARHSP groups [5]. Recently reported dual-transmission of some HSP subtypes makes their molecular characterisation even more complicated. Due to the HSP heterogeneity, next-generation sequencing (NGS) became a highly useful screening tool in HSP investigations and differential diagnosis. Broad NGS studies have revealed a clinical and genetic overlap between different HSP subtypes, as well as between other neurodegenerative disorders, such as hereditary spinocerebellar ataxias (SCAs), amyotrophic lateral sclerosis (ALS) and neuropathies [6].

In the present study, we analysed familial HSP patients through spastic-ataxia spectrum disease genes according to the approach suggested by Synofzik et al. [6].

## Materials and methods

The study was approved by the Bioethics Committee of the Institute of Psychiatry and Neurology in Warsaw. All of the participants provided informed consent.

**Fig. 1:**
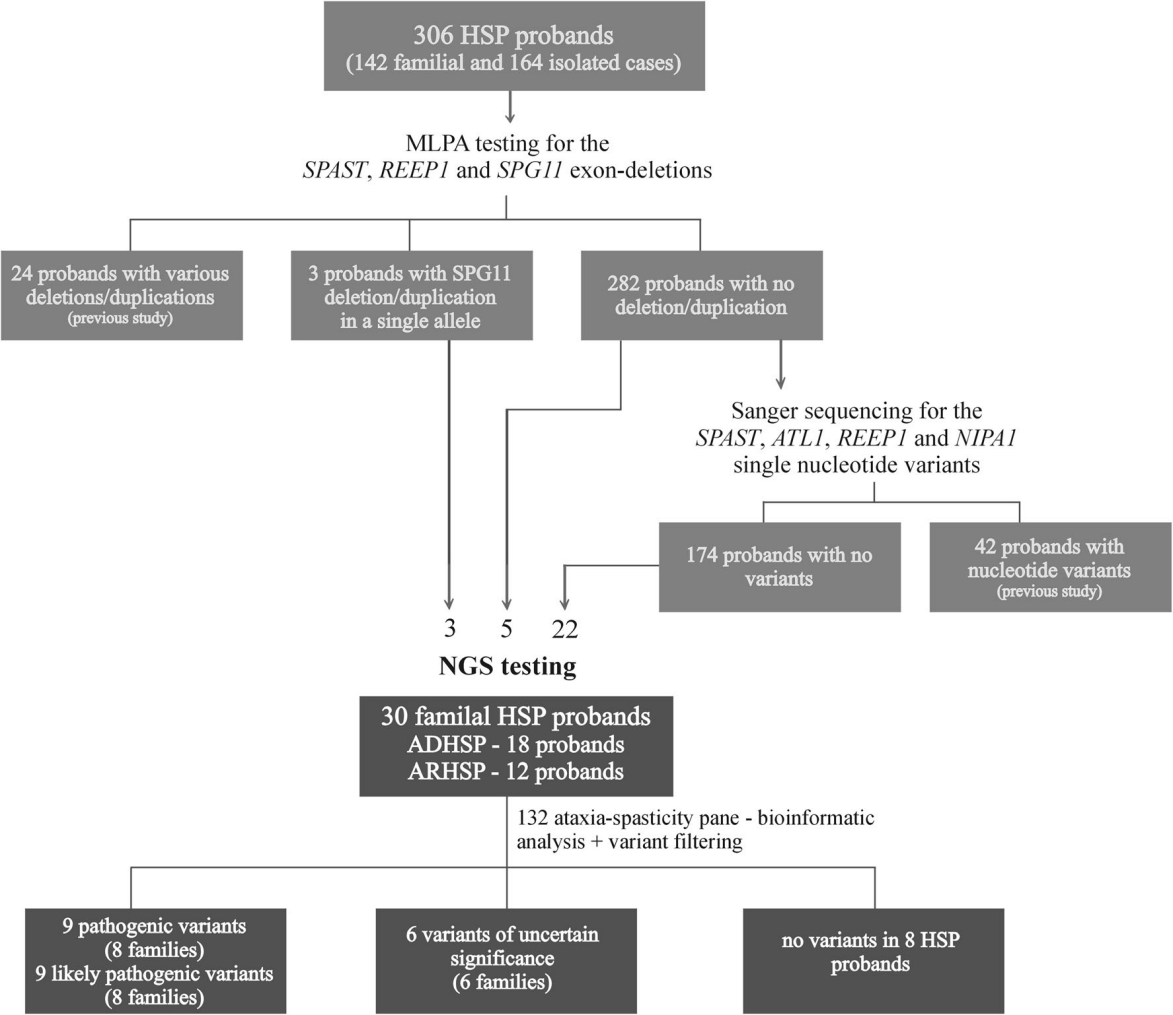
Analysed cohort and methods used during HSP diagnostics. Detailed description of the identified variants is presented in tables

In the presented study, we aimed to test a group of 30 unrelated hereditary spastic paraplegia patients using the targeted Illumina TruSight™ One Sequencing Panel (Illumina). The original HSP cohort comprised 306 probands in which Multiplex Ligation-dependent Probe Amplification (MLPA) and Sanger Sequencing had been performed to diagnose five HSP subtypes (SPG3, SPG4, SPG6, SPG11 and SPG31) in 62 families [7–10]. Out of the remaining 244 probands, 30 familial HSP index cases were selected for NGS testing. The major inclusion criteria comprise: (i) spastic paraplegia as a main clinical feature, (ii) positive family history and (iii) availability of DNA sample for more than one affected family member and/or potential carriers. The families’ history suggested AD inheritance in 18 and AR in 12 families. In three probands, *SPG11* deletions and duplication had been identified in one allele, and NGS sequencing focused on searching for the second causative variant to confirm the AR SPG11. One identified carrier of the *SPAST* pathogenic variant was used as a positive control in the NGS screening (Fig. 1).

All studied patients were evaluated according to the Fink criteria for HSP [11]. The HSP pure form was observed in 16 probands, and the complicated form was observed in 14 probands.

The Illumina TruSight™ One Sequencing Panel covering the coding regions of the 4813 genes associated with the known clinical phenotypes was used (https://www.illumina.com/products/by-type/clinical-research-products/trusight-one.html). The panel includes over 125,000 80-mer probes constructed according to the human NCBI37/hg19 reference genome. The probe set was designed for enrichment of approximately 62,000 exons spanning 4813 genes (https://www.illumina.com/products/by-type/clinical-research-products/trusight-one.html). The library preparation, labelling and enrichment were performed according to the protocol using 50 ng of DNA input. The coding regions of 132 genes linked to spastic paraplegias, hereditary ataxias and related movement disorders were analysed. The data were analysed using Illumina VariantStudio 2.2 and visualised in Integrated Genomics Viewer (IGV) (Broad Institute). To investigate the evolutionary conservation score (PhyloP) and functional prediction of identified mutations, we used SIFT (http://sift.jcvi.org/), Polyphen2 (http://genetics.bwh.harvard.edu/pph2/), MutationTaster (http://www.mutationtaster.org/) and Alamut software (http://www.interactive-biosoftware.com/), as well as the dbSNP (https://www.ncbi.nlm.nih.gov/projects/SNP/) and ClinVar databases (https://www.ncbi.nlm.nih.gov/clinvar/).

NGS data were filtered according to the following criteria: (i) read depth higher than 20 reads and variant frequency higher than 25%; (ii) variants reported less frequently than 0.005 in the Exome Aggregation Consortium database (http://exac.broadinstitute.org/); and (iii) exclusion of all the synonymous and deep intronic variants.

The bioinformatically analysed 132 ataxia-spasticity panel genes involved the following: (1) 37 genes directly linked with HSP: 12-ADHSP, 22-ARHSP and 3-XLHSP; (2) 25 genes linked with hereditary ataxias: 12 AD spinocerebellar ataxia (SCA), 11 ARSCA (SCAR) and four spastic-ataxia (SPAX) genes; (3) three leucodystrophy genes; (4) 14 amyotrophic lateral sclerosis (ALS) genes; (5) 16 genes linked with different neuropathies, including five hereditary motor neuropathies (HMN) and six Charcot Marie-Tooth neuropathies; and (6) other complex movement or multisystem disorders with prominent gait disturbances, comprising 42 genes (Supplementary Table 1). Because certain genes are linked with more than one phenotype, the number of genes and conditions are not equal. The classification and interpretation of the identified variants were performed according to recommendations of the American College of Medical Genetics and Genomic and the Association for Molecular Pathology (ACMGG&AMP) (Table 1) [12]. Variants selected through filtering were confirmed by Sanger sequencing in the probands and their family members.

**Table 1:** Interpretation of all variants identified in HSP probands according to the ACMGG&AMP guidelines [Richards and others 2015]

Patient ID	Gene	cDNA change	ACMG criteria	ACMG classification
SPG0902	*ATL1*	NM_015915.4:c.715C>T	PM1 + PM2 + PP1 + PP3 + PP4 + PP5	Likely pathogenic
		NP_056999.2:p.(Arg239Cys)		
SPG0901	*ATL1*	NM_015915.4:c.1064A>C	PM1 + PM2 + PP3 + PP4	Likely pathogenic
		NP_056999.2:p.(Asn355Thr)		
SPG1301	*SPAST*	NM_014946.3:c.1378C>T	PM1 + PM2 + PP4 + PP3 + PP5	Likely pathogenic
		NP_055761.2:p.(Arg460Cys)		
SPG0102	*SPAST*	NM_014946.3:c.1597G>T	PVS1 + PM2 + PM4 + PM5 + PP4	Pathogenic
		NP_055761.2:p.(Glu533*)		
SPG1401	*SPAST*	NM_014946.3:c.1617-2A>G	PVS1 + PM2 + PP4	Pathogenic
SPG0403	*WASHC5*	NM_014846.3:c.647C>T	PP1 + PP3 + PP4	Uncertain significance
		NP_055661.3:p.(Pro216Leu)		
SPG0302	*WASHC5*	NM_014846.3:c.1859T>C	PM2 + PP1 + PP3 + PP4 + PP5	Likely pathogenic
		NP_055661.3:p.(Val620Ala)		
SPG0201	*KIF5A*	NM_004984.2:c.484C>T	PM1 + PP3 + PP4 + PP5	Likely pathogenic
		NP_004975.2:p.(Arg162Trp)		
SPG1402	*KIF5A*	NM_004984.2:c.1402C>T	PP3 + PP4	Uncertain significance
		NP_004975.2:p.(Arg468Trp)		
SPG1101	*KIF1A*	NM_001244008.1:c.962G>A	PM1 + PM2 + PM4 + PP3 + PP4	Likely pathogenic
		NP_001230937.1:p.(Gly321Asp)		
SPG0601	*SPG11*	NM_025137.3:c.408_428del	PM2 + PM4 + PP4 + PP5	Likely pathogenic
		NP_079413.3:p.(Glu136_Ile143del)		
		NM_025137.3:c.3075insA	PVS1 + PM2 + PP5	Pathogenic
		NP_079413.3:p.(Glu1026Argfs*4)		
SPG1002	*SPG11*	NM_025137.3:c.733_734del	PVS1 + PM2 + PM3 + PP5	Pathogenic
		NP_079413.3:p.(Met245Valfs*2)		
		NM_025137.3:c.1471_1472del	PVS1 + PM2 + PM3 + PP5	Pathogenic
		NP_079413.3:p.(Leu491Aspfs*66)		
		NM_025137.3:c.6632G>A	PP2	Uncertain significance
		NP_079413.3:p.(Arg2211His)		
SPG1003	*SPG11*	NM_025137.3:c.1471_1472del	PVS1 + PM2 + PM3 + PP5	Pathogenic
		NP_079413.3:p.(Leu491Aspfs*66)		
		NM_025137.3:c.3075insA	PVS1 + PM2 + PM3 + PP5	Pathogenic
		NP_079413.3:p.(Glu1026Argfs*4)		
SPG0702	*SPG11*	NM_025137.3:c.1275insA	PVS1 + PM2 + PP4	Pathogenic
		NP_079413.3:p.(Glu426Argfs*3)		
SPG0502	*SPG11*	NM_025137.3:c.1457-2A>G	PVS1 + PM2 + PM3 + PP5	Pathogenic
		NM_025137.3:c.5623C>T	PVS1 + PM2 + PM3 + PP5	Pathogenic
		NP_079413.3:p.(Gln1875*)		
SPG0301	*SPG11*	NM_025137.3:c.2849delT	PVS1 + PM2 + PM4	Pathogenic
		NP_079413.3:p.(Leu950Trpfs*13)		
SPG0103	*SPG11*	NM_025137.3:c.2987_2989del	PM2 + PM4 + PP3 + PP4	Likely pathogenic
SPG0701	CYP27A1	NM_000784.3:c.379C>T	PM2 + PM3 + PP3 + PP5	Likely pathogenic
		NP_000775.1:p.(Arg127Trp)		
SPG0303	*ITPR1*	NM_001168272.1:c.2687C>T	PP1 + PP3	Uncertain significance
		NP_001161744.1:p.(Ala896Val)		
SPG0401	*ITPR1*	NM_001168272.1:c.2687C>T	PP1 + PP3	Uncertain significance
		NP_001161744.1:p.(Ala896Val)		
SPG1203	*ITPR1*	NM_001168272.1:c.3412A>G	PP3	Uncertain significance
		NP_001161744.1:p.(Met1138Val)		
		NM_001168272.1c.6304G>T	PP3	Uncertain significance
		NP_001161744.1:p.(Ala2102Ser)		
SPG0503	*SETX*	NM_015046.5:c.7417C>G	PP1 + PP3	Uncertain significance
		NP_055861.3:p.(Leu2473Val)		

*PVS* very strong evidence of pathogenicity, *PS* strong evidence of pathogenicity, *PM* moderate evidence of pathogenicity, *PP* supporting evidence of pathogenicity

## Results

The NGS TruSight™ One output data reached approximately 97% of the aligned reads. A mean number of 16,752,119 reads with 259 base pair length fragments per sample was obtained. An average of 91.2% of targeted reads passed the Q score, whereas 88% were covered at least 30 times.

In this study, we identified 18 pathogenic and likely pathogenic variants in 16 spastic paraplegia probands, as well as six variants of uncertain significance (Table 2; Table 3). The most frequent HSP genetic types, SPG4 and SPG3, were identified in five probands: *SPAST* (SPG4) pathogenic variants in three probands and *ATL1* (SPG3) in two probands. In four of the mentioned probands, a previous study involved only the MLPA screening, and one of the SPG4 patients was known to carry a pathogenic variant. In 11 out of 22 individuals, in whom *SPAST, ATL1* and *REEP1* gene single nucleotide variants (SNV) were previously excluded by Sanger sequencing, we identified three HSP subtypes with AD transmission: *WASHC5* (SPG8), *KIF5A* (SPG10) and *KIF1A* (SPG30) and *SPG11* (SPG11) as the only ARHSPs. Moreover, in one case, a homozygous variant in the *CYP27A1* gene, known as pathogenic in cerebrotendinous xanthomatosis (CTX), was identified. Among six variants of uncertain significance we detected: *WASHC5*, *KIF5A, SETX* and *ITPR1* variants in families with AD mode of inheritance. We were not able to detect any variant corresponding to phenotype in 27% of the examined cohort (four cases with AD and four with AR mode of inheritance).

**Table 2:** Pathogenic and likely pathogenic variants identified in spastic paraplegia probands

Patient ID	Gene	Chr	Genomic position	cDNA change (protein change)	Allele zygosity	PhyloP score	ClinVar	SIFT/PolyPhen/MutTaster	ExAC allele frequency†	rs number	Inheritance
SPG0902	*ATL1*	14	g.51080061	NM_015915.4:c.715C>T	ht	1208	Pathogenic	del/ps_dam/dc	0	rs119476046	AD
				NP_056999.2:p.(Arg239Cys)							
SPG0901	*ATL1*	14	g.51089911	NM_015915.4:c.1064A>C	ht	4,81		del/ps_dam/dc	0	na	AD
				NP_056999.2:p.(Asn355Thr)							
SPG1301	*SPAST*	2	g.32362002	NM_014946.3:c.1378C>T	ht	2754	Pathogenic	delet/pb_dam/dc	0	rs878854990	AD
				NP_055761.2:p.(Arg460Cys)							
SPG0102	*SPAST*	2	g.32368465	NM_014946.3:c.1597G>T	ht	5131		na/na/dc	0	na	AD
				NP_055761.2:p.(Glu533*)							
SPG1401	*SPAST*	2	g.32370004	NM_014946.3:c.1617-2A>G (splice acceptor variant)	ht	3963		na/na/dc	0	na	AD
SPG0302	*WASHC5*	8	g.126069814	NM_014846.3:c.1859T>C	ht	5107		tol/ps_dam/dc	0	na	AD
				NP_055661.3:p.(Val620Ala)							
SPG0201	*KIF5A*	12	g.57958739	NM_004984.2:c.484C>T	ht	1838	na	del/pb_dam/dc	0 (0.0000083)	rs748551786	AD
				NP_004975.2:p.(Arg162Trp)							
SPG1101	*KIF1A*	2	g.241713675	NM_001244008.1:c.962G>A	ht	5425		del/pb_dam/dc	0	na	AD
				NP_001230937.1:p.(Gly321Asp)							
SPG0601	*SPG11*	15	g.44952643	NM_025137.3:c.408_428del	c_ht	1.28‡	Pathogenic	na/na/dc	0	rs312262714	AR
				NP_079413.3:p.(Glu136_Ile143del)							
			g.44905697	NM_025137.3:c.3075insA		na	Pathogenic	na/na/dc	0.0000083 (0.0000083)	rs312262752	
				NP_079413.3:p.(Glu1026Argfs*4)							
SPG1002	*SPG11*	15	g. 44949427	NM_025137.3:c.733_734del	c_ht	0.23‡	Pathogenic	na/na/dc	0.000045 (0.000107)	rs312262720	AR
				NP_079413.3:p.(Met245Valfs*2)							
			g. 44941193	NM_025137.3:c.1471_1472del		2.12‡	Pathogenic	na/na/dc	0.0000083 (0.0000083)	rs312262727	
				NP_079413.3:p.(Leu491Aspfs*66)							
			g.44859744	NM_025137.3:c.6632G>A		0.952	us	tol/bn/dc	0.00127 (0.0008)	rs144165094	
				NP_079413.3:p.(Arg2211His)							
SPG1003	*SPG11*	15	g.44941193	NM_025137.3:c.1471_1472del	c_ht	2.12‡	Pathogenic	na/na/dc	0.0000083 (0.0000083)	rs312262727	AR
				NP_079413.3:p.(Leu491Aspfs*66)							
			g.44905697	NM_025137.3:c.3075insA		na	Pathogenic	na/na/dc	0.0000083 (0.0000083)	rs312262752	
				NP_079413.3:p.(Glu1026Argfs*4)							
SPG0702	*SPG11*	15	g.44943869	NM_025137.3:c.1275insA	c_ht	na		na/na/dc	0	na	AR
				NP_079413.3:p.(Glu426Argfs*3)							
				c.(4906 + 1_4907–1)_ (5121 + 1_5122–1)del (deletion of exon 29§)		na		na			
SPG0502	*SPG11*	15	g.44941211	NM_025137.3:c.1457-2A>G (splice acceptor variant)	c_ht	3652	Pathogenic	na/na/dc	0	rs312262726	AR
			g.44876255	NM_025137.3:c.5623C>T		0.705	Pathogenic	na/na/dc	0.00006 (0.000041)	rs141848292	
				NP_079413.3:p.(Gln1875*)							
SPG0301	*SPG11*	15	g.44907749	NM_025137.3:c.2849delT	c_ht	3361		na/na/dc	0	na	AR
				NP_079413.3:p.(Leu950Trpfs*13)							
				c.(1735 + 1_1736–1)_ (2244 + 1_2245–1)del (deletion of exons 9–11§)		na		na			
SPG0103	*SPG11*	15	g.44907609	NM_025137.3:c.2987_2989del	c_ht	1.96‡		na/na/dc	0	na	AR
				NP_079413.3:p.(Cys996del)							
				c.(4743 + 1_4744–1)_ (5121 + 1_5122–1)dup (duplication of exons 28–29§)		na		na			
SPG0701	CYP27A1	2	g.219674423	NM_000784.3:c.379C>T	hm	1529	Pathogenic	del/pb_dam/dc	0 (0.000025)	rs201114717	AR
				NP_000775.1:p.(Arg127Trp)							
			g.219674423	NM_000784.3:c.379C>T							
				NP_000775.1:p.(Arg127Trp)							

† according European (non-Finnish) population; total frequency in bracket; ‡ average PhyloP score for each deleted base pair; § MLPA testing result

*bn* benign, *c_ht* compound heterozygous, *dc* disease causing, *del* deleterious, *ht* heterozygous, *hm* homozygous, *na* not applicable, *pb_dam* probably damaging, *pol* polymorphism, *ps_dam* possibly damaging, *rs* reference SNP, *tol* tolerated, *us* uncertain significance

**Table 3:** Variants of uncertain significance found in spastic paraplegia probands

Patient ID	Gene	Chr	Genomic position	cDNA change	Allele zygocity	PhyloP score	ClinVar	SIFT/PolyPhen/MutTaster	ExAC allele frequency†	rs number	Inheritance
SPG0403	*WASHC5*	8	g.126091044	NM_014846.3:c.647C>T	ht	5443	na	tol/pb_dam/dc	0.001694 (0.00122)	rs72720524	AD
				NP_055661.3:p.(Pro216Leu)							
SPG1402	*KIF5A*	12	g.57965883	NM_004984.2:c.1402C>T	ht	1.19	na	del/bn/dc	0 (0.0000084)	rs771021589	AD
				NP_004975.2:p.(Arg468Trp)							
SPG0303	*ITPR1*	3	g.4716885	NM_001168272.1:c.2687C>T	ht	1719	us	tol/bn/dc	0.00051 (0.000315)	rs201519806	AD
				NP_001161744.1:p.(Ala896Val)							
SPG0401	*ITPR1*	3	g.4716885	NM_001168272.1:c.2687C>T	ht	1719	us	tol/bn/dc	0.00051 (0.000315)	rs201519806	AD
				NP_001161744.1:p.(Ala896Val)							
SPG1203	*ITPR1*	3	g.4725441	NM_001168272.1:c.3412A>G	na	4274	us	tol/bn/dc	0.0008452 (0.000484)	rs199698357	AD
				NP_001161744.1:p.(Met1138Val)							
			g.4821291	NM_001168272.1c.6304G>T		4331		del/bn/dc	0.000105 (0.000058)	rs373973399	
				NP_001161744.1:p.(Ala2102Ser)							
SPG0503	*SETX*	9	g.135140243	NM_015046.5:c.7417C>G	ht	3436	na	tol/ps_dam/pol	0 (0.000033)	rs760196991	AD
				NP_055861.3:p.(Leu2473Val)							

† according European (non-Finnish) population, total frequency in bracket

*bn* benign, *dc* disease causing, *del* deleterious, *ht* heterozygous, *na* not applicable, *pb_dam* probably damaging, *pol* polymorphism, *ps_dam* possibly damaging, *rs* reference SNP, *tol* tolerated, *us* uncertain significance

### Autosomal dominant HSPs

#### *ATL1* (SPG3)

One known pathogenic *ATL1* variant: c.715C>T (p.Arg239Cys) and one novel: c.1064A>C (p.Asn355Thr) were identified in two HSP probands. The variants presented pure HSP with the age of onset at the first and second years of life.

#### *SPAST* (SPG4)

In the *SPAST* gene, the variants were identified in three probands: a missense (c.1378C>T-p.Arg460Cys), nonsense (c.1597G>T-p.Glu533*) and splice site (c.1617-2A>G) mutation. *SPAST* c.1378C>T is a known pathogenic variant, a moderately conserved nucleotide and highly conserved amino acid position. The two other *SPAST* gene variants (c.1597G>T and c.1617-2A>G) have not been previously described, neither in the patient cohorts nor in population studies. The ages at onset in the three SPG4 patients were 35, 42 and 28 years, respectively. Two probands had pure HSP, while in one with the nonsense variant, a complicated HSP phenotype with neuropathy as an additional symptom was observed.

#### *WASHC5* (SPG8)

The *WASHC5* missense variants were found in two HSP probands and at least one affected individual within their families. Patient SPG0302 was found to have *WASHC5* c.1859C>T (p.Val620Ala). The female proband and her affected sibling—aged 39 and 37 years at onset—had frontal cortex atrophy. Moreover, in patient SPG0302, white matter and thoracic spinal cord lesions were present. The male proband SPG0403, with *WASHC5* c.647C>T (p.Pro216Leu), presented a complex HSP with dysarthria. His brother with the same variant had intellectual disability in addition to HSP (but he had a verified birth asphyxia—a possible cause of the brain damage).

#### *KIF5A* (SPG10)

Two *KIF5A* variants were identified in two probands. One of them, *KIF5A* c.484C>T (p.Arg162Trp), which localised in motor domain of the kinesin protein was present in a proband with pure HSP and onset of symptoms at age 41. The second, *KIF5A* variant c.1402C>T (p.Arg468Trp), which altered the stalk part of the protein, was identified in a female proband with pyramidal signs, ataxia, dysdiachokinesia, bradykinesia, titubation, ophthalmoparesis and dementia, in whom first symptoms appeared after turning 40. In MRI, marked atrophy of the cerebellum and cerebral cortex (predominantly temporal and parietal) was observed.

#### *KIF1A* (SPG30)

A heterozygous *KIF1A* c.962G>A (p.Gly321Asp) variant, localised in the motor domain of the protein, was found in an AD pedigree. The female proband and her mother had childhood onset, complex hereditary spastic paraplegia and cognitive decline.

### Autosomal recessive HSPs

#### *SPG11* (SPG11)

The NGS analysis enabled us to identify ten different *SPG11* variants (with the ExAC frequency below 0.005) in seven probands. In all of them, the variants were present in both alleles. In the SPG1002 proband, three different variants were detected. In three other patients with single variants found in this study, SPG0103, SPG0301 and SPG0702, the microrearrangements: duplication of exons 28–29, deletions of exons 9–11 and exon 29, respectively, were localised in trans. Five of the variants were frameshift deletions or insertions, two were in-frame deletions, one was in the splice-site, one was nonsense and one was a missense change. In SPG1002, the missense variant was identified in cis with the frameshift one.

All of the seven SPG11 probands had a complicated form of HSP and showed cognitive impairment: dysarthria 5/7; dysphagia 2/7; nystagmus 3/7; ophthalmoparesis (horizontal gaze) 2/7; cervical dystonia 1/7 and mild ataxia 3/7. In neuroimaging performed in six probands, thin corpus callosum was found in 5/6, periventricular white matter lesions were found in 4/6, and mild cortical and subcortical atrophy was identified in 2/6. EMG provided evidence of polyneuropathy in three out of five examined probands.

#### *CYP27A1* (CTX)

In one proband, NGS revealed a homozygous variant, c.379C>T (p.Arg127Trp) in the *CYP27A1* gene, known as pathogenic in cerebrotendinous xanthomatosis (CTX). The carrier status (heterozygosity) was confirmed in the proband’s father. The patient, with pyramidal and cerebellar signs, petit mal seizures, bilateral cataract and retinal degeneration in the right eye, was classified as a case of the complicated HSP. Mild cortical and subcortical atrophy were present in brain MRI. Furthermore, in the patient’s medical history, vitamin B12 deficiency and nephrolithiasis were documented. To date, neither xanthomas nor other signs characteristic for CTX were not observed in the patient.

### Genes with uncertain significance in HSPs

#### *ITPR1* (GLSP/SCA15/SCA29)

**Fig. 2:**
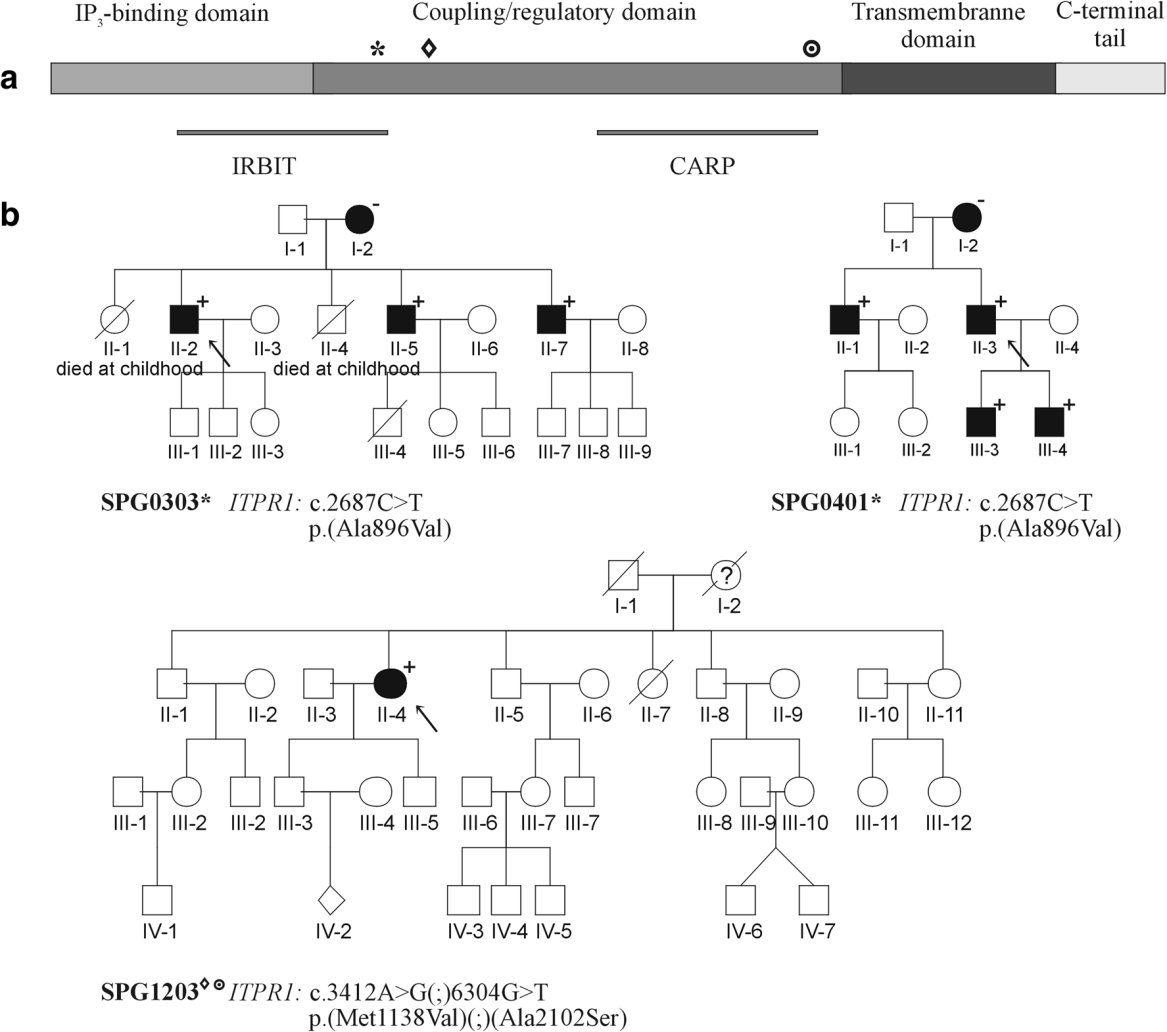
**A** ITPR1 protein scheme. Localization of three identified variants interrupting coupling/regulatory domain is showed by: “*”, p.(Ala896Val); “♦”, p.(Met1138Val); “•”, p.(Ala2102Ser). IRBIT, Inositol 1,4,5-trisphosphate (IP_3_) receptor binding domain; CARP, Carbonic anhydrase–related protein (CA8) binding domain. **B** Pedigrees of three families with *ITPR1* variants. Families SPG0303 and SPG0401 are marked with “*” which indicates *ITPR1*: c. 2687C>T (p.Ala896Val); family SPG1203 is marked with “♦” and “•” which indicate *ITPR1*: c.3412A>G (p.Met1138Val) and c.6304G>T (p.Ala2102Ser), respectively. The “+” points out family members, in whom the DNA samples were tested; “-“affected individuals without DNA testing

Three different variants of uncertain significance were identified in the ADHSP patients. *ITPR1*: c.2687C>T (p.Ala896Val) was identified in seven individuals from two unrelated families with pure HSP. In the SPG1203 proband, two different *ITPR1* variants (c.3412A>G-p.Met1138Val and c.6304G>T-p.Ala2102Ser) were found. A female patient with weakness and spasticity of her lower limbs, balance disturbances and polyneuropathy had onset of symptoms at age 50. Genetic testing in her relatives was impossible; however, her family history may indicate AD inheritance. All the pedigrees and localization of identified *ITPR1* variants are shown in Fig. 2.

#### *SETX* (ALS4/SCAR1)

One *SETX* missense variant of uncertain significance, c.7417C>G (p.Leu2473Val), was detected in a 2-year-old proband and the father, who has been affected since childhood. The father’s neurological examination showed upper and lower limb weakness and spasticity with increased tendon reflexes and clonus.

## Discussion

Due to heterogeneity, the increasing number of involved genes and varieties of phenotypes (disorders) linked to a single gene, the classification and diagnostics of HSPs are challenging. To overcome these difficulties, different NGS approaches have been applied in a number of studies, mostly targeted sequencing but also whole exome sequencing [13–17]. In the present study, we analysed 30 HSP index cases using the Illumina TruSight™ One NGS sequencing panel. Bioinfomatic analysis was performed for 132 out of the 4813 genes included in the panel. This methodology allowed us to identify 25 variants in nine genes. The pathogenic and likely pathogenic variants were identified in 16 probands. In five of them, in whom only MLPA technique had been used for microrearrangement searching, we identified three *SPAST* and two *ATL1* variants by NGS. It is an evidence that MLPA is not sufficient for SPG4 testing alone, nonetheless together with NGS is now a standard in diagnostic approach. Less frequent HSP subtypes were identified in a group of patients in whom the *SPAST*, *ATL1* and *REEP1* pathogenic variants had been previously excluded. Two different variants were identified in *WASHC5* (SPG8, OMIM #603563, previously known as *KIAA0196*) and *KIF5A* (SPG10, OMIM #604187) genes, both regarded as rare HSP subtypes (approximate frequency 1–2%) that may be associated with pure or complicated HSP phenotypes [4]. The *WASHC5*: c.1859T>C (p.Val620Ala) variant has previously been detected in pure HSP patients but has not been reported in either ExAC or the 1000 Genomes projects [18]. The *KIF5A*:c.484C>T (p.Arg162Trp) variant has been reported in a three-generation pedigree with spastic paraplegia as a primary symptom [19].

KIF1A is a neuron-specific motor protein involved in intracellular transport along microtubules. Variants in the *KIF1A* gene have been described in patients with AR hereditary sensory and autonomic neuropathy type 2 (HSAN2, OMIM #614213) and subtype 30 of the hereditary spastic paraplegia (SPG30, OMIM #610357) [20–23]. De novo *KIF1A* variants with AD transmission have been identified in multiple cases with childhood onset of intellectual disability and a number of neurological signs, such as progressive spastic paraplegia, optic nerve atrophy, peripheral neuropathy and cerebral and/or cerebellar atrophy, have been variously classified as autosomal dominant mental retardation type 9 (MRD9, OMIM#614255) [24–28] or complicated hereditary spastic paraplegia [25, 29, 30]. Finally, *KIF1A* mutations have been found in pure HSP subjects [30–32]. In the present study, a dominant *KIF1A* variant localised in the motor domain of the protein was found in a female proband and her mother with childhood onset complex HSP and cognitive decline. Twenty-three out of 25 heterozygous *KIF1A* variants (including the present study) alter the highly conserved motor domain of the protein. However, two out of four variants responsible for recessive HSP and any of the variants identified in HSAN2 are localised in the motor domain. This suggests that localization of the *KIF1A* variants within the gene is not adequate evidence for phenotype transmission. Moreover, the latest data indicate that dominant conditions, including ADHSP, linked with *KIF1A* variants are more frequent than recessive ones.

SPG11 (OMIM #604360) is the only known recessive HSP subtype identified in this study. Contrary to other studies, we have not detected any affected patient with *CYP7B1* (SPG5, OMIM #270800) or *SPG7* (SPG7, OMIM #607259) mutations, or any mutation carriers [13–17, 32]. Moreover, variants in *ZFYVE26* (SPG15, OMIM #270700), which occur with frequency below 0.005 in the ExAC database, were not detected in our cohort.

In addition to the recessive variants, in one case, we detected a homozygous variant in the *CYP27A1* gene. Pathogenic variants in the cytochrome P450 *CYP27A1* gene result in the production of a defective sterol 27-hydrolase enzyme and have been linked with cerebrotendinous xanthomatosis (CTX) (OMIM #213700). Clinical manifestation of CTX includes neurological dysfunction (e.g. cerebellar ataxia, pyramidal signs, and seizures), cataracts, tendon xanthomas and chronic diarrhoea [33, 34]. However, some atypical presentation of symptoms may occur. For example, Verrips et al. described seven patients with *CYP27A1* variants and slowly progressive spinal cord syndrome classified as spinal xanthomatosis. Moreover, similar to our case, all of the patients presented pyramidal signs, and in five of them, spinal cord white matter lesion have been demonstrated. Six out of seven cases studied by Verrips et al. did not have tendon xanthomas [35]. Patients with *CYP27A1* variants affected with pure and complicated HSP but without xanthomas were also described by Burguez et al. and Nicholls et al. [15, 36]. These findings suggest that patients with *CYP27A1* variants may present the broader clinical spectrum including HSP phenotype, nonetheless the lack of the typical symptoms of CTX, especially xantomas, should not exclude the investigation of *CYP27A1* gene mutations.

Variants of uncertain significance within *ITPR1* and *SETX* genes were detected in four cases. *ITPR1* variants have already been described as possibly corresponding to four different phenotypes: multi-exon deletions in *ITPR1* gene to spinocerebellar ataxia type 15 (SCA15, OMIM #606658), single nucleotide variants to spinocerebellar ataxia type 29 (SCA29, OMIM #117360) or ataxic cerebral palsy (Ataxic CP), and the truncated and splice-site variants in Gillespie Syndrome (GLSP, OMIM #206700) also presented ataxia and balance disturbances [37–42]. *ITPR1* encodes a homotetramer calcium channel protein that modulates intracellular calcium signalling. Its primary structure consists of three major domains [43]. In this study the *ITPR1* c.2687C>T (p.Ala896Val) variant was detected in two unrelated families and segregates with pure HSP phenotype in seven cases. We also identified two different *ITPR1* variants in a patient with pyramidal signs and polyneuropathy. Although the three described variants were reported in the ExAC database, their frequency was lower than 0.005 (Table 2b). The relatively mild HSP symptoms in our patients were first observed in adulthood i.e. the age of onset was not optimal for control studies. The segregation data in the families with c.2687C>T (p.Ala896Val) supports its pathogenicity; however, according to the ACMGG&AMP guidelines, this is not adequate evidence to classify it as a pathogenic/probably pathogenic variant. Variants identified in the present study are localised in the coupling-domain and comprise the first report assigning *ITPR1* variants to HSP.

A variant classified as of uncertain significance was also found in the senataxin gene. *SETX* variants are responsible for AR spinocerebellar ataxia (SCAR1) and AD amyotrophic lateral sclerosis (ALS4) [44–48]. The heterozygous variant of the *SETX* gene has also been described as a cause of hereditary motor neuropathy (dHMN) [49, 50]. Taniguchi et al. reported a family with a *SETX* variant misdiagnosed as a hereditary spastic paraplegia [51]. The mentioned variant (*SETX*:c.8C>T) was localised in the N-terminal end of the protein, different than the *SETX*: c.7417C>G (p.Leu2473Val), altering the C-terminal part of the protein, which was identified during our study in father and son with pure HSP. It is localised in the region of the helicase domain, where known pathogenic variants correlated with ALS4 and SCAR1 phenotypes had been reported as well [52].

Although the molecular investigation of rare heterogenic disorders, such as hereditary spastic paraplegias, will soon be based on massive NGS technology, their molecular aetiology assessment still remains challenging. Two major difficulties to face at present are: (1) interpretation of the detected variants (pathogenic vs benign) and (2) classification of the identified variant and its association with a specific disease. Unified and reliable sequence variants interpretation guidelines were developed by the American College of Medical Genetics and Genomics and the Association for Molecular Pathology. Each rare or novel variant should be evaluated in a patient’s and family’s history context, and physical examination and previous differential diagnosis should be performed. Such clinical evaluation is supportive during the process of variants classification as disease-causing, incidental or benign findings [12]. Variants classified as pathogenic but also likely pathogenic have sufficient evidence to be used in genetic counselling and clinical decision-making. In contrast, variants of uncertain significance need further investigation that may result in their reclassification [12].

Implementing NGS technologies in clinical practice also brings problems due to the genotype-phenotype correlation and variants’ classification. The classification systems were designed according to a predominant disease phenotype and/or a mode of inheritance. Currently, various genes corresponding to numerous complex phenotypes, such as spinocerebellar ataxias, spastic paraplegias and amyotrophic lateral sclerosis, are associated with *SPG7*, *SPG11*, *PNPLA6*, *KIF1C* and *SETX*, and they may be inherited as both autosomal dominant and recessive traits *(KIF1A*, *REEP2*, *AFG3L2*, *SETX).* In clinical practice, it becomes problematic whether the identified gene variant should be classified as corresponding to a new phenotype or if it “fits” the patient’s genotype consistent with the previous clinical diagnosis. Synofzik et al. proposed introducing the unbiased modular phenotyping approach to replace the ataxias and hereditary spastic paraplegia classification [6]. In parallel, we also recommend simultaneously testing and analysing the HSP, SCA and ALS genes due to their overlapping phenotype and common cellular pathways involved.

In this paper, we report 24 different variants of nine genes in HSP patients. Seven of the variants are novel. They were classified according to the ACMGG&AMP guidelines, and nine were classified as pathogenic, nine as likely pathogenic and six as of uncertain significance. Among nine analysed genes, five have already been known as directly associated with HSP. NGS testing revealed genetic variants in 22 out of 30 tested families. Altogether with the previous study [8], seven different HSP subtypes have been diagnosed in the Polish group of patients to date. Our data also support the evidence that *KIF1A* (SPG30) variants are more frequent in patients with ADHSP, although they were primarily identified as ARHSP. Moreover, we believe that *CYP27A1* variants should be considered to be complicated HSP phenotype cases, as well.

The overlapping phenotypes of HSP, SCA and ALS are associated with multiple genes; therefore, NGS-based screening provides the best comprehensive genetic diagnostic approach. The most challenging interpretation of the novel variants requires the entire body of clinical and molecular evidence available in the entire studied group of patients sharing a defined spectrum of clinical signs.

## Electronic supplementary material

Supplementary Table 1 (DOCX 20 kb)
