# Supplementary material for: Next-generation sequencing study reveals the broader variant spectrum of hereditary spastic paraplegia and related phenotypes
Source: Neurogenetics. 2019 Feb 19;20(1):27–38. doi: 10.1007/s10048-019-00565-6 (PMC6411833; doi:10.1007/s10048-019-00565-6)
Supplement: Supplementary file 1 — (DOCX 20 kb) [file 10048_2019_565_MOESM1_ESM.docx]

Supplementary table 1. List of the genes included in the bioinformatically analysed panel

| Gene | Phenotype | Phenotype MIM number | Mode of inheritance |
| --- | --- | --- | --- |
| *ABCB7* | ASAT | 301310 | XL |
| *ABCD1* | ALD | 300100 | XL |
| *ABHD12* | PHARC | 612674 | AR |
| *AFG3L2* | SPAX5 / SCA28 | 614487 / 610246 | AR / AD |
| *AIMP1* | HLD3 | 260600 | AR |
| *ALDH18A1* | SPG9 | 601162 | AD |
| *ALS2* | ALS2 | 205100 | AR |
| *ANG* | ALS9 | 611895 | AD |
| *ANO10* | SCAR10 | 613728 | AR |
| *AP4B1* | SPG47 | 614066 | AR |
| *AP4E1* | SPG51 | 613744 | AR |
| *AP4M1* | SPG50 | 612936 | AR |
| *AP4S1* | SPG52 | 614067 | AR |
| *AP5Z1* | SPG48 | 613647 | AR |
| *APTX* | AOA1 | 208920 | AR |
| *ARG1* | Argininemia | 207800 | AR |
| *ATL1* | SPG3 | 182600 | AD |
| *ATP13A2* | SPG78 | 617225 | AR |
| *ATP7A* | DSMAX | 300489 | XL |
| *ATP8A2* | CAMRQ4 | 615268 | AR |
| *AUH* | MGA1 | 250950 | AR |
| *BSCL2* | SPG17 | 270685 | AD |
| *C12orf65* | SPG55 | 615035 | AR |
| *C19orf12* | SPG43 | 615043 | AR |
| *CCDC88C* | SCA40 | 616053 | AD |
| *CCT5* | Neuropathy, hereditary sensory with spastic paraplegia | 256840 | AR |
| *CHMP2B* | ALS17 | 614696 | AD |
| *CLCN2* | LKPAT | 615651 | AR |
| *CLN5* | CLN5 | 256731 | AR |
| *CYP27A1* | CTX | 213700 | AR |
| *CYP7B1* | SPG5 | 270800 | AR |
| *DARS2* | LBSL | 611105 | AR |
| *DNM2* | CMTDIB | 606482 | AD |
| *DNMT1* | ADCADN | 604121 | AD |
| *ELOVL4* | SCA34 | 133190 | AD |
| *ENTPD1* | SPG64 | 615683 | AD |
| *ERLIN2* | SPG18 | 611225 | AR |
| *FA2H* | SPG35 | 612319 | AR |
| *FAM126A* | HLD5 | 610532 | AR |
| *FARS2* | SPG77 | 617046 | AR |
| *FGF14* | SCA27 | 609307 | AD |
| *FIG4* | ALS11 / CMT4J | 612577 / 611228 | AD / AR |
| *FLVCR1* | AXPC1 | 609033 | AR |
| *FUS* | ALS6 | 608030 | AD |
| *GAD1* | SPSQ1 | 603513 | AR |
| *GALC* | Krabbe disease | 245200 | AR |
| *GAN* | GAN1 | 256850 | AR |
| *GARS* | HMN5A / CMT2D | 600794 / 601472 | AD |
| *GBA* | Gaucher disease type II and III | 230900 / 231000 | AR |
| *GBE1* | APBN | 263570 | AR |
| *GCH1* | DRD | 128230 | AD / AR |
| *GFAP* | ALXDRD | 203450 | AD |
| *GJC2* | SPG44 | 613206 | AR |
| *GLB1* | GM1-gangliosidosis type II | 230600 | AR |
| *GRM1* | SCAR13 | 614831 | AR |
| *HACE1* | SPPRS | 616756 | AR |
| *HEXA* | GM2-gangliosidosis type I | 272800 | AR |
| *HSPB1* | HMN2B / CMT2F | 608634 / 606595 | AD |
| *HSPB3* | HMN2C | 613376 | AD |
| *HSPB8* | HMN2A / CMT2L | 158590 / 608673 | AD |
| *HSPD1* | SPG13 | 605280 | AD |
| *IGHMBP2* | DSMA1 / CMT2S | 604320 / 616155 | AR |
| *ITPR1* | SCA15 / SCA29 | 606658 / 117360 | AD |
| *KANK1* | SPSQ2 | 612900 | AD |
| *KCNC3* | SCA13 | 605259 | AD |
| *KCND3* | SCA19 | 607349 | AD |
| *KIF1A* | SPG30 | 610357 | AR |
| *KIF5A* | SPG10 | 604187 | AD |
| *L1CAM* | SPG1 | 303350 | XL |
| *LYST* | CHS | 214500 | AR |
| *MARS2* | SPAX3 | 611390 | AR |
| *MTHFR* | Homocystinuria due to MTHFR deficiency | 236250 | AR |
| *MTPAP* | SPAX4 | 613672 | AR |
| *NEFH* | CMT2CC | 616924 | AD |
| *OPHN1* | Mental retardation, X-linked with cerebellar hypoplasia | 300486 | XL |
| *OPTN* | ALS12 | 613435 | AR |
| *PANK2* | NBIA1 | 234200 | AR |
| *PDYN* | SCA23 | 610245 | AD |
| *PEX7* | RCDP1 | 215100 | AR |
| *PFN1* | ALS18 | 614808 | AD |
| *PHYH* | Refsum disease | 266500 | AR |
| *PLA2G6* | NBIA2B | 610217 | AR |
| *PLEKHG5* | DSMA4 / CMTRIC | 611067 / 615376 | AR |
| *PLP1* | SPG2 | 312920 | XL |
| *PMPCA* | SCAR2 | 213200 | AR |
| *PNPLA6* | SPG39 | 612020 | AR |
| *POLG* | SANDO | 607459 | AR |
| *PQBP1* | RENS1 | 309500 | XL |
| *PRKCG* | SCA14 | 605361 | AD |
| *PSEN1* | AD3 | 607822 | AD |
| *RAB3GAP1* | WARBM2 | 614225 | AR |
| *RAB3GAP2* | SPG69 | 609275 | AR |
| *REEP1* | SPG31 | 610250 | AD |
| *RTN2* | SPG12 | 604805 | AD |
| *RUBCN* | SCAR15 | 615705 | AR |
| *SACS* | SACS | 270550 | AR |
| *SETX* | SCAR1 / ALS4 | 606002 / 602433 | AR / AD |
| *SIGMAR1* | DSMA2 / ALS16 | 605726 / 614373 | AR |
| *SIL1* | MSS | 248800 | AR |
| *SLC16A2* | SPG22 | 300523 | XL |
| *SLC25A15* | Hyperornithinemia – hyperammonemia – homocitrullinuria syndrome | 238970 | AR |
| *SLC33A1* | SPG42 | 612539 | AD |
| *SLC9A6* | MRXSCH | 300243 | XL |
| *SOD1* | ALS1 | 105400 | AD |
| *SOX10* | PCWH | 609136 | AD |
| *SPAST* | SPG4 | 182601 | AD |
| *SPG11* | SPG11 | 604360 | AR |
| *SPG20* | SPG20 | 275900 | AR |
| *SPG21* | SPG21 | 248900 | AR |
| *SPG7* | SPG7 | 607259 | AR |
| *SPR* | Dopa-responsive dystonia due to sepiapterin reductase deficiency | 612716 | AD / AR |
| *SPTBN2* | SCA5 / SCAR14 | 600224 / 615386 | AD / AR |
| *SYNE1* | SCAR8 | 610743 | AR |
| *SYT14* | SCAR11 | 614229 | AR |
| *TARDBP* | ALS10 | 612069 | AD |
| *TDP1* | SCAN1 | 607250 | AR |
| *TGM6* | SCA35 | 613908 | AD |
| *TH* | Segawa syndrome | 605407 | AR |
| *TPP1* | SCAR7 | 609270 | AR |
| *TRPV4* | HMN8 / HMSN2C | 600175 / 606071 | AD |
| *TTBK2* | SCA11 | 604432 | AD |
| *TXN2* | COXPD29 | 616811 | AR |
| *UBQLN2* | ALS15 | 300857 | XL |
| *UCHL1* | SPG78 | 617225 | AR |
| *VAPB* | ALS8 / SMAFK | 608627 / 182980 | AD |
| *VCP* | ALS14 / CMT2Y | 613954 / 616687 | AD |
| *VLDLR* | CAMRQ1 | 224050 | AR |
| *WASHC5* | SPG8 | 603563 | AD |
| *WDR73* | GAMOS | 251300 | AR |
| *WWOX* | SCAR12 | 614322 | AR |
| *ZFYVE26* | SPG15 | 270700 | AR |
| *ZFYVE27* | SPG33 | 610244 | AD |

**AD** – Alzheimer disease; **ADCADN** – automal dominant cerebellar ataxia, deafness and narcolepsy; **ALD** – adrenoleukodystrophy; **ALS** – amyotrophic lateral sclerosis; **ALXDRD** – Alexander disease; **AOA** – ataxia with oculomotor apraxia; **APBN** – polyglucosan body neuropathy, adult form; **ASAT** – sideroblastic anemia with ataxia; **AXPC1** – posterior column ataxia with retinitis pigmentosa; **CAMRQ** – cerebellar ataxia, mental retardation and disequilibrum syndrome; **CHS** – Chediak-Higashi syndrome; **CLN** – neuronal ceroid lipofuscinosis; **CMT** – Charcot-Marie-Tooth disease; **CMTDIB** – Charcot-Marie-Tooth disease, dominant intermediate B; **CMTRIC** - Charcot-Marie-Tooth disease, recessive intermediate C; **COXPD** – combined oxidative phosphorylation deficiency; **CTX** – cerebrotendinous xanthomatosis; **DSMA** – distal spinal muscular atrophy; **DSMAX** – distal spinal muscular atrophy, X-linked; **DRD** – dopa-recponsive dystonia; **GAMOS** – Galloway-Mowat Syndrome; **GAN** – giant axonal neuropathy ; **HLD** – hypomielinating leucodystrophy; **HMN** – distal hereditary motor neuropathy; **HMSN** – hereditary motor and sensory neuropathy; **LBSL** – leucencephalopathy with brainstem and spinal cord involvement and lactate elevation; **LKPAT** – leukoencephalopathy with ataxia; **MGA1** – 3-methylglutaconic aciduria, type1; **MRXSCH** – X-linked syndromic mental retardation, Christianson type; **MSS** – Marinesco-Sjogren syndrome; **NBIA** – neurodegeneration with brain ion accumulation; **SACS** – spastic ataxia Charlevoix-Saguenay; **SANDO** – sensory ataxic neuropathy, dysarthria and ophtalmoparesis; **SCA** – spinocerebellar ataxia; **SCAN** – spinocerebellar ataxia, autosomal recessive with axonal neuropathy; **SCAR**- spinocerebellar ataxia, autosomal recessive; **SMAFK** – late onset spinal muscular atrophy, Finkel type; **SPAX** – spastic ataxia; **SPG** – spastic paraplegia; **SPPRS**- spastic paraplegia and psychomotor retardation with or without seizures; **SPSQ** – cerebellar palsy, spastic quadriplegic; **PCWH** – peripheral demyelinating neuropathy, central dysmyelination, Waardenburg syndrome and Hirschsprung disease; **PHARC** – polyneuropathy, hearing loss, ataxia, retinitis pigmentosa and cataract; **RCDP1** – rhizomelic chondrodysplasia punctata; **RENS** – Renpenning syndrome; **WARBM** – Warburg micro syndrome;
